# Fish Meal Replacement with a Combination of Meat Meal and Chicken Byproduct Meal on Growth Performance, Feed Utilization, Biochemical Parameters and Muscle Composition of Juvenile Red Seabream (*Pagrus major*)

**DOI:** 10.3390/ani15111581

**Published:** 2025-05-28

**Authors:** Buddhi E. Gunathilaka, Seong-Mok Jeong, Byung-Hwa Min, Jinho Bae, Sang-Woo Hur, Sang-Guan You, Sang-Min Lee

**Affiliations:** 1 Department of Aquatic Life Medicine, Gangneung–Wonju National University, Gangneung 25457, Republic of Korea; glbeg44@gwnu.ac.kr; 2 Aquafeed Research Center, National Institute of Fisheries Science, Pohang 37517, Republic of Korea; smjeong1@korea.kr (S.-M.J.);; 3 Department of Marine Food Science and Technology, Gangneung–Wonju National University, Gangneung 25457, Republic of Korea; umyousg@gwnu.ac.kr

**Keywords:** meat meal, chicken byproduct meal, alternative protein sources, fish meal replacement, red seabream

## Abstract

A combination of meat meal (MM) and chicken byproduct meal (CBM) were evaluated as fish meal (FM) replacers in the diets of juvenile red seabream (*Pagrus major*). The control diet was formulated with 60% FM (CON). Four other diets were designed by reducing the FM levels to 45, 30, 15, and 0% by adding 14, 28, 42, and 56% MM and CBM in a 1:1 ratio to compensate for the reduced protein level (designated as MC14, MC28, MC42, and MC56, respectively). Red seabream (averaging 4.57 g) were distributed among 15 fiberglass tanks (40 fish/tank) and assigned to one of three replicates of the diet. After an eight-week long feeding trial, the growth performance of the fish fed the CON, MC14, and MC28 diets were comparable with the control group, and were significantly higher than the MC42 and MC56 groups. Feed utilization was significantly higher in the MC28 group compared to the other groups. The lowest growth and feed utilization were observed in the MC56 group. Serum lysozyme and SOD activities were significantly reduced when the dietary FM level decreased. Plasma total cholesterol levels were significantly reduced when the FM level was decreased in the diets, exhibiting a significant linear trend. The CON group exhibited a significantly higher cholesterol level compared to the MC42 and MC56 groups. The muscle amino acid profile was not significantly affected. The muscle myristic acid (14:0) was significantly decreased, while the palmitic acid (16:0) level increased with the increase of MM and CBM in the diets. Therefore, the results indicate that the combination of MM and CBM can be used to replace 50% of the FM from the red seabream diets without negative influences compared to a diet containing 60% FM.

## Simple Summary

Red seabream is a marine carnivore species widely cultured in Northeast Asia. Protein supplementation for carnivorous fish is a critical task in the aquafeed sector due to the limitation of marine-origin protein sources. Meat meal and chicken byproduct meals are alternative protein sources used in aquafeeds. Both ingredients contain high levels of animal protein and are reported to replace some extent of the fish meal from the diets for carnivorous fish species. In the present study, we evaluated different fish meal replacement levels with a combination of meat meal and chicken byproduct meal in the diets for juvenile red seabream (*Pagrus major*). After a feeding trial, we observed that growth performance and feed utilization of red seabream were not significantly influenced when the diets contain 30% fish meal with meat meal and chicken byproduct meal. Muscle fatty acid profiles were slightly altered. Immune parameters were gradually decreased with the decrease of the dietary fish meal level. Overall, a combination of MM and CBM reduced the fish meal level in the diets to 30% without negative influences compared to a diet containing 60% FM.

## 1. Introduction

Alternative protein sources are utilized to formulate aquafeed due to the insufficient supply of fish meal (FM) that is considered to be the principal protein source. Different protein sources and their combinations have been evaluated to replace the FM in the fish diets during the last two decades. Alternative protein sources can be categorized into two main groups: plant-based or animal-based protein ingredients. Animal-based protein ingredients, primarily produced from terrestrial farm animal byproducts, insects, and microorganisms, are used in diets for aquaculture species [1,2,3].

Meat meal (MM) is manufactured using the remaining inedible flesh after meat processing. MM has been utilized in low-FM diets for aquaculture species for several decades. Especially, the FM in carnivorous fish diets was successfully reduced using MM without sacrificing the fish performance. According to recent studies, the FM level in olive flounder diets (*Paralichthys olivaceus*) was reduced to 26% with 22% MM while maintaining growth performance, feed utilization, immunity, and disease resistance [4]. The nutrient digestibility of MM in olive flounder was comparable or even greater to that of the conventional FM [5]. The FM level was reduced to 21% with 9% porcine MM in golden pompano diets (*Trachinotus blochii*) without significant impacts on growth, antioxidant status, intestinal health, and gut *Photobacterium* count [6]. In rock fish (*Sebastes schlegeli*) diets, approximately 18% FM was reduced with MM [7]. Zaman et al. [8] reduced 20% of FM with MM from the olive flounder diet containing 65% FM. In another study, approximately 50% of the FM was replaced from the pacific white shrimp (*Litopenaeus vannamei*) diet with porcine MM [9], highlighting the potential of MM as a viable alternative protein source in aquafeed formulations.

Chicken byproduct meal (CBM) is prepared using side streams, including heads, necks, feet, and viscera, that are not intended for human consumption [10,11]. It also has a high protein content with a desirable quantity of other nutrients making it a valuable feed ingredient for aquafeed formulations [10,11,12]. CBM is less expensive than FM and remains consistently available year-round due to its high production rate. Consequently, CBM has been used successfully as a substitute for FM in the diets designed for many aquaculture species.

Red seabream (*Pagrus major*) is a marine carnivorous fish species, belonging to the family Sparidae, cultured in the Asian region including Japan and the Republic of Korea. FM is an important protein source for red seabream aquaculture. However, studies on the feed and nutrition of red seabream revealed that FM in diets can be replaced with different other protein sources, including soy protein concentrate, soybean meal, krill meal, protein hydrolysates, and insect meal, with additional functional ingredients, i.e., taurine and amino acids [13,14,15]. MM and CBM were tested in the diets for fish species belonging to the Sparidae family. Sabbagh et al. [16] reported that FM in the gilthead seabream diet (*Sparus aurata*) was substituted up to 100% with CBM compared to a control diet containing 36% FM. They observed similar growth, feed utilization, expression of structural protein, and muscle composition. Later, Fontinha et al. [17] recorded that 83% of FM could be replaced with CBM from the gilthead seabream diet without affecting growth and gut microbiota. CBM replaced 30% of the FM from the black seabream diet (*Acanthopagrus schlegelii*) improving the growth performance and the expression of genes related to protein metabolism [18]. In sobaity seabream diets (*Sparidentex hasta*), 45% of FM was replaced by CBM maintaining growth, protein digestibility, digestive enzyme activities, and biochemical indices. MM was earlier reported as a potential substitute of FM in non-FM diets for red seabream [19]. It was later used as an ingredient in the FM-reduced diets for red seabream [20]. Murashita et al. [21] reported that CBM, along with other animal protein sources, stimulates bile secretion in red seabream after oral administration. They also stated an improvement in intestinal enzyme activity in red seabream fed CBM. In our previous studies, MM and CBM were tested in the red seabream diet combined with plant protein sources, and reduced the FM level from 60% to 40% without sacrificing the growth performance [22]. We also observed a mixture of MM and CBM at 1:1 ratio replaced 50% of FM, from 60% to 30%, improving the growth performance and feed utilization of red seabream compared to other protein sources and their combinations [23].

The present study was conducted as a follow up of our previous study [23] to evaluate the proper FM replacement level with the 1:1 combination of MM and CBM in the diets for juvenile red seabream through the effects on growth performance, feed utilization, plasma biochemical indices, muscle amino acid profile, and muscle fatty acid profile.

## 2. Materials and Methods

### 2.1. Experimental Diets

Five experimental diets were formulated with approximately 49% and 13% crude protein and lipids, respectively (Table 1). The diets were designed following our previous study [23] with some modifications. A diet formulated to contain 60% FM and 6% soybean oil was considered as the control (CON). Four other diets were designed by reducing the FM levels to 45, 30, 15, and 0% by adding 14, 28, 42, and 56% MM and CBM in a 1:1 ratio to compensate for the reduced protein levels (designated as MC14, MC28, MC42, and MC56, respectively). Soybean oil in the diets were gradually replaced with fish oil when the FM amount was decreased in the diets to maintain a similar level of marine originated lipids in each diet. Methionine and lysine were also added to compensate for the reduced amount when the FM was replaced with MM and CBM in each diet. After measuring, all dry ingredients were thoroughly mixed with oil and 30% distilled water to make a dough. Then, the dough was passed through a noodle machine (SP-50, Gum Gang Engineering, Daegu, Republic of Korea) crushed into ideal size (3–5 mm) and dried at 40 °C for 12 h. Dry diets were stored at −20 °C until use. The fatty acid and amino acid profiles of all five diets are provided in Table 2 and Table 3, respectively.

### 2.2. Experimental Fish Feeding Trial and Conditions

Red seabream were purchased from a private hatchery (Uljin-gun, Republic of Korea). The feeding trial was conducted at the Gangneung–Wonju National University, Marine Biology Center. They were fed a commercial diet and acclimatized in fiber-glass circular tanks for two weeks. Then, juveniles, averaging 4.57 ± 0.03 g, were placed at a density of 40 fish per tank in 15 tanks (300 L). The tanks were randomly assigned to three replicates of five formulated diets. Then, the fish in each tank were daily fed the assigned diets until satiation at 09:10 and 17:10 h for eight weeks. The photoperiod was controlled with fluorescent lights to provide the natural day length. The water temperature ranged between 17.7 and 21.3 °C. Also, the salinity, pH and dissolved oxygen level were monitored every day and ranged between 32.1 and 33.4 ppt, 7.47 and 7.87, and 6.57 and 7.29 mg/L, respectively, during the feeding trial. There were no notable differences in water quality parameters among the experimental tanks.

### 2.3. Sample Collection and Analysis

The fish in each experimental tank were starved for 24 h at the end of the eight-week feeding trial. Then, the fish were caught carefully with a fish catching net while counting. The bulk fish weight of each tank was measured for the calculation of weight gain (WG), specific growth rate (SGR), feed intake (FI), feed efficiency (FE), and protein efficiency ratio (PER). Twelve fish from each tank were randomly selected and anesthetized with 2-phenoxyethanol (200 ppm). Blood samples were collected from the caudal vein of the twelve captured fish. Blood samples from six fish were withdrawn to separate plasma for biochemical analyses and from the remaining six fish to separate serum samples for immune parameter analyses. Blood samples were withdrawn with heparinized syringes to prevent clotting before the plasma separation. Plasma and serum samples were separated by centrifugation at 5000× *g* for 10 min, pipetted out to new vials and stored at −80 °C. Blood was kept at room temperature for 30 min to facilitate clotting before separation of the serum samples. The remaining 12 fish, after blood sampling, were stored to freeze at −20 °C for proximate composition analyses of muscle, amino acids, and fatty acid levels. Then, the remaining fish in each tank were killed with a high dose of 2-phenoxyethanol (500 ppm). The weight and length of each fish was measured to the closest 0.1 mm to determine condition factor (CF). Each fish was dissected, and the viscera and liver were collected to measure weight for calculation of hepatosomatic (HSI) and viscerosomatic indices (VSI).

The moisture and ash levels of the feed ingredients, diets, and the fish muscle were analyzed after drying at 125 °C for 6 h in a dry oven, and at 550 °C for 6 h in a muffle furnace, respectively, according to standard methods as explained by the AOAC [24]. The crude protein level was analyzed, using 0.5 g of samples after digesting with concentrated sulfuric acid (95%), distilling with 8 M sodium hydroxide, recovering nitrogen with boric acid, and titrating with 0.05 M sulfuric acid, with a Kjeltec Analyzer (Buchi, Flawil, Switzerland). The crude lipid level of the samples was determined after boiling 0.5 g of each sample in 60 mL ethyl ether in a Soxhlet extractor (VELP Scientifica, Usmate Velate, Italy). The muscle and diet fatty acid profiles were determined using a gas chromatographic method with a PerkinElmer Clarus 600 gas chromatograph (PerkinElmer, Shelton, CT, USA) as followed by Sankian et al. [25], and calculated as a percentage of total fatty acid level in each sample. The amino acid composition of both diet and muscle samples were analyzed after properly controlled acid hydrolysis (6 N HCL reflux for 23 h at 110 °C) using a High-speed Amino Acid Analyzer (L-8800, Hitachi, Tokyo, Japan) at the Marine Biology Regional Center, Gangneung, Republic of Korea. The lysozyme activity was measured after mixing 20 μL of serum samples with 100 μL of lyophilized *Micrococcus lysodeikticus* (Sigma-Aldrich, St. Louis, MO, USA) as a substrate based on a turbidimetric technique following Khosravi et al. [26]. The serum superoxide dismutase activity (SOD) was measured in 20 μL of serum samples by analyzing the inhibition rate of tetrazolium with the SOD enzyme in the serum using a commercial Assay Kit (19,160, Sigma-Aldrich, St. Louis, MO, USA). A blood analyzer, FUJI DRI-CHEM NX500i, Fujifilm, Minato, Japan, was utilized to measure the plasma biochemical parameters including glutamic-oxaloacetic transaminase, alkaline phosphatase, total cholesterol, triglycerides, glutamate-pyruvate transaminase, total protein, albumin, and glucose levels.

### 2.4. Statistical Analysis

Each experimental tank was considered to be a statistical unit. Data collected from the statistical units were analyzed using one-way ANOVA. The significance of differences in the mean effects of the diets was determined using Duncan’s [27] multiple range test with SPSS version 20.0 (SPSS Inc., Chicago, IL, USA). The Shapiro–Wilk, and the Levene tests were applied for the verification of homogeneity of the observed data. A follow-up trend analysis was performed using orthogonal polynomial contrasts to determine whether the effect is linear and/or quadratic. Statistical significance was determined at *p* < 0.05. Data were presented as a mean ± standard deviation. Arcsine transformation was performed on percentage data prior to statistical analysis.

## 3. Results

### 3.1. Growth Performance, Feed Utilization and Survival

The results of growth performance and feed utilization are presented in Table 4. The FBW and WG of the fish were significantly higher in the CON, MC14, and MC28 groups compared to those fish fed the MC42 and MC56 diets (*p* < 0.05). Both parameters are comparable among the CON, MC14, and MC28 groups. The MC56 group showed significantly lower FBW, SGR, and WG than the fish fed the MC42 diet (*p* < 0.05). The SGR was significantly greater in the MC28 group compared to the MC42 and MC56 groups (*p* < 0.05). The SGR values calculated in the CON and MC14 groups were comparable with the MC28 and MC42 groups, while all four groups exhibited significantly higher values than the MC56 group (*p* < 0.05). Feed intake (FI) was significantly higher in the CON and MC14 groups compared to the other groups (*p* < 0.05). The FE and PER were also significantly improved in the MC28 group than in all the other groups (*p* < 0.05). However, both the FE and PER of the CON, MC14, and MC42 group were comparable and significantly higher than those of the MC56 group (*p* < 0.05). The FBW, WG, SGR, and PER exhibited both significant linear and quadratic trends with the increase of MM and CBM in the diets (*p* < 0.05). The FI showed only a significant linear trend. The survival rate of the fish was not significantly affected by the dietary treatments.

### 3.2. Non-Specific Immune Responses

The results of the lysozyme and SOD analyses are presented in Table 5. The lysozyme activity was significantly greater in the CON and MC14 groups compared to that of the fish fed the MC42 diet (*p* < 0.05). The results of the MC28 and MC56 groups were comparable with those of the other groups. The SOD activity of the MC14 and MC28 groups were significantly higher than the fish fed the MC42 diet, while the CON and MC56 groups exhibited comparable results to all the other groups (*p* < 0.05). Both lysozyme and SOD activities exhibited significant linear trends with the decrease of FM in the diets (*p* < 0.05).

### 3.3. Plasma Biochemical Parameters

The plasma biochemical parameters are provided in Table 6. The total cholesterol level was significantly higher in the fish fed the CON diet compared to that of the MC42 and MC56 groups (*p* < 0.05). The fish fed the MC14 and MC28 diets exhibited cholesterol levels comparable to those of the other groups. Additionally, the plasma total cholesterol levels were reduced when the FM level decreased in the diets, exhibiting a significant linear trend (*p* < 0.05). Other parameters were not significantly influenced by the dietary MM and CBM.

### 3.4. Muscle Proximate, Fatty Acid and Amino Acid Compositions

The muscle proximate, fatty acid, and amino acid compositions of red seabream are presented in Table 7, Table 8, and Table 9, respectively. The muscle proximate composition was not significantly altered by the experimental diets. Myristic acid (14:0) in the fish fed the CON diet was significantly higher than that of the MC56 diet (*p* < 0.05). By contrast, the palmitic acid (16:0) level of the CON group was significantly lower than the MC42 and MC56 groups (*p* < 0.05). Docosanoic acid (22:0) was not detected in the MC56 group. Eicosatrienoic acid (20:3 n-3) was also not detected in the MC28, MC42, or MC56 groups. Other fatty acid levels in the fish muscle were not significantly affected by the dietary treatments. Muscle amino acid levels were also not significantly modified by the dietary treatments although the Ile and Ser levels exhibited significant linear trends with the decrease of FM in the diets (*p* < 0.05).

### 3.5. Biometric Parameters

Biometric parameters are presented in Table 10. The CF, HSI, and VSI were also not significantly affected by the dietary treatments.

## 4. Discussion

The growth performance and feed utilization of the fish fed the MC28 diet were comparable with the CON diet, indicating that the 1:1 mixture of MM and CBM can be used to replace 30% of FM from the control diet without sacrificing the fish performance. Similarly, the best performance was observed when the fish were fed the diet containing the 30% MC mixture in our previous study [23]. Essential fatty acids, such as EPA and DHA levels, were decreased in the diets with the increased levels of MM and CBM (Table 2). These fatty acids are important to maintain the proper growth of fish species including red seabream. In early studies, the EPA and DHA requirements of early juvenile red seabream were estimated to be 2.25 and 0.95%, respectively [28]. For juvenile red seabream (3 g), it was reported to be 1% EPA and 0.5% DHA [29]. Sarker et al. [30] reported that red seabream (51 g) can synthesize EPA and DHA from their precursors. However, it was recently documented that red seabream were unable to synthesize both EPA and DHA due to lack of some important enzymes required in the process [31]. The precursors of EPA and DHA, i.e., alpha-linolenic acid (18:3 n-3) levels, were also lower in the MC42 and MC56 diets (Table 2), although we increased the fish oil level in the diets. Consequently, the maximum amount of both EPA and DHA were provided with only the CON, MC14, and MC28 diets in the present study, explaining a reason for the high growth rates compared to the MC42 and MC56 diets. Moreover, several essential amino acid levels were reduced with the increase of MM and CBM in the diets, even though the methionine and lysine levels were approximately similar in all the diets. Balanced amino acid levels in diets improve the FI and FE [32], especially for the functional ingredients, i.e., taurine, known as a conditionally essential amino acid for fish, that are important to improve the growth performance, FI, and FE of red seabream fed low-FM diets [14]. In the present study, the low availability of essential amino acids in the MC42 and MC56 diets might have led to the lower growth in the fish. Therefore, the addition of more EPA, DHA, amino acids, and functional ingredients can be recommended for further FM replacement using over 30% MM and CBM in the red seabream diet.

The serum lysozyme and SOD activities were reduced in the red seabream fed the MC42 and MC56 diets, indicating that these activities were significantly decreased with the increase of MM and CBM levels in the diets while decreasing the FM level. In our previous study, the lysozyme activity was significantly improved in the fish fed the diet containing the 30% MC mixture compared to several other alternative protein sources [23]. Lysozyme is an important enzyme to eliminate invading cells in animals [33]. SOD is also an important antioxidant enzyme that indirectly induces the immunity of the fish [34]. In red seabream, both the lysozyme and SOD activities were reported to decrease when the fish were fed the low-FM diets [26,35]. Especially high levels of terrestrial animal protein in diets negatively impact on the fish immunity. For instance, the expression of inflammation-related genes was upregulated in hybrid grouper (*Epinephelus fuscoguttatus*♀ × *Epinephelus lanceolatus*♂) fed diets having a high level of CBM [36]. The activities of several innate immune parameters in sunshine bass (*Morone chrysops* × *M. saxatilis*) were decreased by a diet containing high levels of CBM [37]. Both antioxidant activity and immune-related gene expression of juvenile barramundi (*Lates calcarifer*) were decreased when all FM in the diets was replaced with CBM [38]. However, Subhadra et al. [39] observed no significant effects on the lysozyme activity of largemouth bass (*Micropterus salmoides*) with diets containing different oil sources with similar FM levels. In our previous study, we assumed that the proper nutrient status of the fish fed the MC diet was the reason for the improved lysozyme activity [23]. Therefore, more studies are required to evaluate the effect of dietary MM and CBM on the immune responses of red seabream focusing on the protein and lipid sources in the diets.

The plasma cholesterol level of red seabream was significantly reduced with the increase of MM and CBM levels in the diets. In our previous study, these biochemical indices were not significantly influenced by different protein sources when the diets contained 30% FM with MM and CBM [23]. The blood cholesterols level of Nile tilapia (*Oreochromis niloticus*) fed diets containing over 50 g/kg CBM were significantly lower than the high FM control group [40]. This suggested that the increase of monounsaturated and polyunsaturated fatty acids (MUFA and PUFA) with CBM supplementation was the reason for the observation because similar results were reported when the fish oil in the yellowtail kingfish diet (*Seriola lalandi*) was replaced with poultry oil [41]. In the present study, the PUFA level was decreased with the increase of MM and CBM levels in the red seabream diets, although the MUFA level was increased with the reduced FM levels. High levels of dietary MUFA increased the plasma cholesterol level of rats [42]. The experimental diets in the present study were formulated to contain increasing levels of fish oil and decreasing levels of soybean oil (Table 1) to balance the marine originated lipid level. Norambuena et al. [43] reported that low cholesterol intake resulted in high plasma cholesterol level in rainbow trout (*Oncorhynchus mykiss*). Therefore, we assumed that the cholesterol level in the red seabream was decreased with the decrease of FM and soybean oil or with increase of MM and CBM in the diets. However, the cholesterol level of juvenile cobia (*Rachycentron canadum*) was not significantly affected by dietary CBM supplementation at the expense of FM [44]. Therefore, future evaluations are required to further confirm these assumptions.

Muscle amino acid and proximate compositions were not significantly influenced by dietary MM or CBM supplementation in the present study. The lysine and methionine levels of the MC diets were higher than the CON diet because both amino acids were supplemented with the diets. However, the experimental diets contained decreasing levels of several essential amino acids, such as histidine, isoleucine, phenyl alanine, and threonine (Table 2). In line with Gunathilaka et al. [23], we assumed that the growth of red seabream was retarded rather than altering the muscle composition with available amino acids because the growth performance of the fish was reduced with the increase of MM and CBM in the diets. Supportively, similar results were observed in red seabream fed a dipeptide form of phenylalanine [45], and in European seabass (*Dicentrarchus labrax*) fed insect meals [46]. However, the trend was not followed by red seabream fed plant protein sources and microalgae in non-FM diets [47]. Therefore, the relation between dietary and muscle amino acid composition should be investigated in future studies. In the case of the fatty acid composition of the muscle, our results showed that myristic acid (14:0) in the fish was significantly decreased, while the palmitic acid (16:0) level was increased significantly with the increasing MM and CBM levels while decreasing the FM in the diets. These observations reflected the composition of both fatty acids in the diets. However, levels of other fatty acids were not significantly different among treatments regardless of the dietary fatty acid levels. Accordingly, we believed that fish growth was limited to the fatty acids that were available in the diets. However, Seong et al. [48] reported significantly changed fatty acid levels in the whole-body samples of red seabream fed plant protein and micro algae. They also analyzed the liver fatty acid composition and observed the same trend as observed in whole-body samples. It is well documented that the amounts of fatty acids in diets are reflected in the fatty acid content of the fish liver as excess fatty acids are stored in the liver [49]. The viscera and liver fatty acids levels should be examined and compared with the muscle fatty acid levels of red seabream fed MM and CBM containing diets in future studies to understand the results of the present study. Moreover, Chen et al. [50] observed significantly different muscle fatty acid levels in largemouth bass fed different lipid sources. Therefore, lipid sources might also affect the muscle fatty acid levels. In this study, we fed fish with decreasing levels of FM and increasing levels of fish oil. Soybean oil levels were reduced when the FM level decreased in the diets. The combinations of lipid sources might balance the fatty acid availability to observe significantly unchanged results in the present study.

The CF and organosomatic indices were also not significantly changed by the FM replacement with MM and CBM in the present study. CF is known as the weight–length relationship of fish. Low CF indicates the low muscle growth of an individual fish compared to its length. VSI and HSI indicate the weight of viscera or liver compared to the fish body. The low values represent small-sized viscera or liver compared to the high values. In the present study, the lowest CF, VSI, and HSI were observed in the MC56 group, indicating retarded growth due to the FM replacement. The addition of fish oil was not sufficient to alleviate the effects of the FM replacement. However, the results observed in the dietary groups were not significantly different. We assumed that the growth of the fish was restricted due to the availability of essential nutrition in the experimental diets used in this study.

## 5. Conclusions

In summary, the results indicate that a 1:1 mixture of MM and CBM can be used to replace 50% of the FM without compromising growth performance, feed utilization, and innate immunity of red seabream when compared to a diet containing 60% FM. Further FM replacement retard fish performances even after adding marine-originated lipids in diets. The findings of this study are important for future studies to improve the efficiency of low FM diets containing MM and CBM for red seabream.

## Figures and Tables

**Table 1:** Formulation and proximate composition of the experimental diets for red seabream (*Pagrus major*) (%, dry matter basis).

Ingredients	CON	MC14	MC28	MC42	MC56
Fish meal ^1^	60.0	45.0	30.0	15.0	0.00
Meat meal ^2^		7.00	14.0	21.0	28.0
Chicken byproduct meal ^3^		7.00	14.0	21.0	28.0
Corn gluten	5.00	5.00	5.00	5.00	5.00
Wheat flour	19.00	19.45	19.9	20.35	20.8
Squid liver powder	6.00	6.00	6.00	6.00	6.00
Fish oil		1.50	3.00	4.50	6.00
Soybean oil	6.00	4.50	3.00	1.50	0.00
Vitamin premix ^4^	1.50	1.50	1.50	1.50	1.50
Mineral premix ^5^	2.00	2.00	2.00	2.00	2.00
Choline chloride	0.50	0.50	0.50	0.50	0.50
Methionine		0.30	0.60	0.90	1.20
Lysine		0.25	0.50	0.75	1.00
Proximate composition					
Dry matter	95.1	94.2	93.9	94.6	93.2
Crude protein	49.9	48.5	49.3	49.0	51.1
Crude lipids	13.2	13.6	13.9	13.7	13.9
Crude ash	11.6	11.3	10.9	10.5	10.0

^1^ Anchovy fish meal (crude protein, 65.9; crude lipids 9.41). ^2^ SCI Co., Ltd., Hongseong-gun, Chungcheongnam-do, Republic of Korea. ^3^ Daekyung Oil & Transportation Co., Ltd., Busan, Republic of Korea. ^4^ Vitamin mixture composition (unit/kg mix): ascorbic acid, 6400 mg; tocopherol acetate, 37,500 mg; thiamin nitrate, 5000 mg; riboflavin, 10,000 mg; pyridoxine hydrochloride, 5000 mg; nicotinic acid, 37,500 mg; Ca-D-pantothenate, 17,500 mg; inositol, 75,000 mg; biotin, 50 mg; folic acid, 2500 mg; menadione sodium bisulfite, 2500 mg; retinol acetate, 5,000,000 IU; cholecalciferol, 1,000,000 IU; cyanocobalamin, 25 mg; riboflavin, 10,000 mg. ^5^ Mineral mixture composition (g/kg mix); ferrous fumarate, 12.5; manganese sulfate, 11.3, ferrous sulfate, 20; cupric sulfate, 1.25; cobaltous sulfate, 0.75; zinc sulfate, 13.75; calcium iodate, 0.75; magnesium sulfate, 80.2; aluminum hydroxide, 0.75.

**Table 2:** Fatty acid composition of the experimental diets (%, fatty acid) for red seabream (*Pagrus major*).

Fatty Acid	CON	MC14	MC28	MC42	MC56
12:0	0.05	0.05	0.07	0.08	0.11
14:0	2.07	1.88	1.75	1.61	1.50
14:1	0.00	0.03	0.07	0.09	0.10
15:0	0.39	0.31	0.25	0.20	0.14
16:0	15.9	16.7	17.7	18.8	20.3
16:1	2.47	2.49	2.54	2.59	2.66
17:0	0.40	0.37	0.34	0.32	0.29
18:0	5.64	5.94	6.33	6.78	7.41
18:1 n-9	25.5	28.3	31.1	34.1	37.7
18:2 n-6	30.4	28.8	26.9	24.7	21.9
20:0	0.37	0.34	0.32	0.29	0.28
20:1	0.83	1.07	1.11	1.14	1.21
18:3 n-3	3.26	3.27	3.17	3.01	2.73
20:2	0.13	0.12	0.14	0.06	0.07
22:0	0.36	0.34	0.32	0.33	0.35
20:3, n-6	0.13	0.11	0.10	0.13	0.14
22:1 n-9	0.17	0.17	0.18	0.19	0.00
20:3 n-3	0.05	0.04	0.04	0.04	0.00
23:0	0.72	0.63	0.54	0.47	0.33
24:0	0.22	0.19	0.17	0.15	0.14
20:5 n-3	3.29	2.78	2.25	1.70	0.98
24:1	0.45	0.38	0.35	0.30	0.22
22:6 n-3	7.13	5.70	4.28	2.92	1.31
∑SFA	26.2	26.7	27.8	28.9	30.8
∑MUFA	29.5	32.4	35.3	38.3	41.8
∑n3FA	13.7	11.8	9.75	7.68	5.02
∑n6FA	30.5	28.9	27.0	24.9	22.0
∑HUFA	10.6	8.63	6.67	4.79	2.43
∑n3/∑n6	0.45	0.41	0.36	0.31	0.23

**Table 3:** Amino acid composition of soy protein concentrate (%, total amino acid).

Amino Acid	CON	MC14	MC28	MC42	MC56
Essential amino acids					
Arg	5.62	5.36	5.44	5.97	6.14
His	3.60	5.43	4.34	3.26	3.00
Ile	8.03	3.77	3.33	3.22	2.76
Met	2.99	3.91	4.16	3.98	4.43
Leu	8.23	4.30	7.34	7.54	6.66
Lys	8.03	9.17	8.01	7.55	7.10
Phe	3.71	3.19	3.06	3.15	2.86
Thr	4.79	4.43	3.98	3.78	3.65
Val	4.90	5.57	4.96	4.48	4.10
Non-essential amino acids					
Ala	6.26	6.87	7.12	7.46	7.78
Asp	9.26	9.02	8.15	7.80	7.12
Cys	2.00	1.28	1.13	0.94	0.87
Glu	15.7	16.4	15.8	15.7	15.6
Gly	5.82	7.01	8.45	9.93	11.6
Pro	5.78	6.38	7.36	8.18	9.67
Ser	4.37	4.55	4.16	4.10	3.87
Tyr	3.27	1.24	1.28	1.25	1.23

**Table 4:** Growth performance and feed utilization and survival of red seabream (*Pagrus major*) fed the five experimental diets for 8 weeks.

	FBW (g) ^1^	WG (%) ^2^	SGR (%/day) ^3^	FI (g/fish) ^4^	FE (%) ^5^	PER ^6^	SUR (%) ^7^
CON	15.8 ± 0.79 ^a^	243 ± 9.4 ^a^	2.20 ± 0.05 ^ab^	16.6 ± 0.93 ^a^	61.3 ± 10.8 ^b^	1.43 ± 0.25 ^b^	81.7 ± 9.46
MC14	15.9 ± 1.59 ^a^	251 ± 31 ^a^	2.24 ± 0.16 ^ab^	16.3 ± 1.42 ^a^	62.7 ± 9.51 ^b^	1.37 ± 0.24 ^b^	80.0 ± 4.33
MC28	16.3 ± 0.93 ^a^	258 ± 37 ^a^	2.27 ± 0.18 ^a^	14.1 ± 0.47 ^b^	81.7 ± 6.04 ^a^	1.55 ± 0.11 ^a^	95.8 ± 1.44
MC42	13.7 ± 0.92 ^b^	199 ± 4.2 ^b^	1.96 ± 0.03 ^b^	14.4 ± 0.79 ^b^	58.4 ± 6.21 ^b^	1.14 ± 0.12 ^b^	86.7 ± 5.20
MC56	10.7 ± 1.00 ^c^	133 ± 10 ^c^	1.51 ± 0.08 ^c^	13.1 ± 0.84 ^b^	40.7 ± 6.57 ^c^	0.82 ± 0.13 ^c^	86.7 ± 10.4
Pr > F *							
ANOVA	<0.000	<0.000	<0.000	0.004	0.002	0.001	0.128
Linear	<0.000	<0.000	<0.000	<0.000	0.012	0.012	0.222
Quadratic	0.003	0.001	<0.000	0.798	0.001	0.001	0.183

Values are the mean of triplicate groups and are presented as a mean ± SD. Values with different superscript letters in the same row are significantly different (*p* < 0.05). The lack of superscript letters indicates no significant differences among treatments. ^1^ Final mean body weight. ^2^ Weight gain percentage = 100 × (final mean body weight − initial mean body weight)/initial mean body weight. ^3^ Specific growth rate = [(log_e_ final body weight − log_e_ initial body weight)/days] × 100. ^4^ Feed intake = dry feed consumed (g)/number of fish. ^5^ Feed efficiency = (fish wet weight gain/feed intake) × 100. ^6^ Protein efficiency ratio = wet weight gain/total protein given. ^7^ Survival rate = (final fish number/initial fish number) × 100. * Significance probability associated with the F-statistic.

**Table 5:** Non-specific immune response of red seabream (*Pagrus major*) fed the five experimental diets for 8 weeks.

	Lysozyme ^1^	SOD ^2^
CON	38.6 ± 1.23 ^a^	59.2 ± 2.67 ^ab^
MC14	38.3 ± 0.45 ^a^	62.8 ± 1.63 ^a^
MC28	37.2 ± 0.86 ^ab^	62.4 ± 2.74 ^a^
MC42	32.7 ± 2.85 ^b^	52.3 ± 2.76 ^b^
MC56	33.7 ± 0.37 ^ab^	55.6 ± 1.24 ^ab^
Pr > F *		
ANOVA	0.050	0.040
Linear	0.008	0.036
Quadratic	0.920	0.263

Values are the mean of triplicate groups and presented as a mean ± SD. Values with different superscript letters in the same row are significantly different (*p* < 0.05). ^1^ Lysozyme activity (µg mL^−1^). ^2^ Superoxide dismutase (% inhibition). * Significance probability associated with the F-statistic.

**Table 6:** Plasma biochemical parameters of juvenile red seabream (*Pagrus major*) fed the five experimental diets for 8 weeks.

	GOT ^1^	GPT ^2^	ALP ^3^	CHO ^4^	TG ^5^	TP ^6^	ALB ^7^	GLU ^8^
CON	38.7 ± 7.84	9.0 ± 1.73	123 ± 26.1	175 ± 25.2 ^a^	183 ± 28.2	2.8 ± 0.21	0.6 ± 0.01	98.7 ± 23.2
MC14	73.3 ± 34.8	15.7 ± 1.76	136 ± 62.2	138 ± 32.7 ^ab^	148 ± 38.9	3.1 ± 0.26	0.8 ± 0.06	121 ± 20.6
MC28	52.0 ± 12.0	14.3 ± 7.88	50.0 ± 11.6	115 ± 32.5 ^ab^	180 ± 83.3	2.3 ± 0.37	0.5 ± 0.13	108 ± 21.4
MC42	74.0 ± 26.9	18.3 ± 4.81	94.3 ± 43.2	95.0 ± 8.62 ^b^	147 ± 45.0	2.8 ± 0.07	0.6 ± 0.03	107 ± 11.2
MC56	75.3 ± 23.1	21.0 ± 6.35	132 ± 30.6	83.7 ± 2.85 ^b^	97.7 ± 18.8	3.1 ± 0.10	0.8 ± 0.06	125 ± 28.0
Pr > F *								
ANOVA	0.730	0.565	0.521	0.049	0.729	0.149	0.133	0.902
Linear	0.336	0.131	0.850	0.013	0.286	0.686	0.512	0.586
Quadratic	0.793	0.892	0.243	0.547	0.619	0.124	0.235	0.971

Values are the mean of triplicate groups and are presented as a mean ± SD. Values with different superscript letters in the same row are significantly different (*p* < 0.05). The lack of superscript letters indicates no significant differences among treatments. ^1^ Glutamic-oxaloacetic transaminase (U/L). ^2^ Glutamate pyruvate transaminase (U/L). ^3^ Alkaline phosphatase (U/L). ^4^ Total cholesterol (mg/dL). ^5^ Triglycerides (ng/dL). ^6^ Total protein (g/dL). ^7^ Albumin (g/dL). ^8^ Glucose (mg/dL). * Significance probability associated with the F-statistic.

**Table 7:** Muscle proximate composition (%, wet basis) of red seabream (*Pagrus major*) fed the five experimental diets for 8 weeks.

	Dry Matter	Crude Protein	Crude Lipids	Crude Ash
CON	24.2 ± 0.39	19.9 ± 0.66	2.31 ± 0.41	1.78 ± 0.14
MC14	24.3 ± 1.10	18.8 ± 0.37	2.11 ± 0.50	1.89 ± 0.22
MC28	24.3 ± 0.14	19.1 ± 0.19	2.07 ± 0.27	1.77 ± 0.02
MC42	24.6 ± 0.77	18.7 ± 0.96	2.05 ± 0.45	1.76 ± 0.23
MC56	24.0 ± 1.26	18.5 ± 0.87	2.03 ± 0.29	1.82 ± 0.14
Pr > F *				
ANOVA	0.912	0.161	0.895	0.881
Linear	0.981	0.065	0.393	0.840
Quadratic	0.520	0.454	0.658	0.978

Values are the mean of triplicate groups and are presented as a mean ± SD. * Significance probability associated with the F-statistic.

**Table 8:** Muscle fatty acid composition (%, fatty acid) of red seabream (*Pagrus major*) fed the five experimental diets for 8 weeks.

						Pr > F *		
	CON	MC14	MC28	MC42	MC56	ANOVA	Linear	Quadratic
14:0	1.22 ± 0.35 ^a^	1.07 ± 0.20 ^ab^	0.85 ± 0.17 ^ab^	1.06 ± 0.16 ^ab^	0.69 ± 0.22 ^b^	0.048	0.033	0.990
15:0	0.27 ± 0.03	0.22 ± 0.01	0.22 ± 0.08	0.23 ± 0.05	0.25 ± 0.03	0.744	0.756	0.227
16:0	17.2 ± 0.35 ^b^	18.1 ± 0.08 ^ab^	18.0 ± 0.63 ^ab^	18.7 ± 0.79 ^a^	18.6 ± 0.69 ^a^	0.047	0.009	0.365
16:1	2.25 ± 0.53	2.11 ± 0.25	1.92 ± 0.36	2.25 ± 0.29	1.95 ± 0.46	0.746	0.559	0.805
17:0	0.31 ± 0.03	0.28 ± 0.02	0.29 ± 0.05	0.25 ± 0.02	0.17 ± 0.15	0.254	0.050	0.384
18:0	7.57 ± 0.45	7.59 ± 0.30	7.62 ± 0.15	7.69 ± 0.61	7.70 ± 0.15	0.990	0.617	0.981
18:1 n-9	24.3 ± 3.22	24.2 ± 2.66	25.8 ± 3.70	25.3 ± 5.25	26.6 ± 3.77	0.929	0.436	0.924
18:2 n-6	24.9 ± 1.04	22.6 ± 2.33	23.6 ± 1.77	20.6 ± 6.01	23.0 ± 1.17	0.560	0.317	0.438
20:0	0.17 ± 0.15	0.22 ± 0.01	0.14 ± 0.12	0.14 ± 0.12	0.14 ± 0.12	0.875	0.514	0.997
18:3 n-6	0.27 ± 0.12	0.17 ± 0.06	0.12 ± 0.11	0.14 ± 0.25	0.11 ± 0.19	0.767	0.274	0.582
20:1	0.93 ± 0.09	0.90 ± 0.06	0.95 ± 0.07	1.03 ± 0.18	0.92 ± 0.07	0.633	0.633	0.626
18:3 n-3	2.08 ± 0.48	1.82 ± 0.29	1.77 ± 0.31	1.54 ± 0.54	1.52 ± 0.26	0.454	0.084	0.749
20:2	0.36 ± 0.05	0.34 ± 0.06	0.61 ± 0.25	0.56 ± 0.30	0.46 ± 0.19	0.441	0.282	0.292
22:0	0.12 ± 0.10	0.05 ± 0.09	0.04 ± 0.08	0.04 ± 0.08	ND	0.568	0.140	0.858
20:3, n-6	0.39 ± 0.06	0.37 ± 0.07	0.40 ± 0.12	0.36 ± 0.10	0.36 ± 0.06	0.971	0.645	0.896
22:1 n-9	0.28 ± 0.01	0.23 ± 0.02	0.25 ± 0.02	0.26 ± 0.03	0.26 ± 0.02	0.155	0.999	0.062
20:3 n-3	0.07 ± 0.07	0.04 ± 0.06	ND	ND	ND			
23:0	0.96 ± 0.33	1.16 ± 0.23	1.15 ± 0.29	1.24 ± 0.42	1.25 ± 0.30	0.828	0.305	0.716
20:5 n-3	3.24 ± 0.76	3.80 ± 1.39	3.17 ± 0.69	5.25 ± 2.40	3.33 ± 0.70	0.357	0.527	0.460
24:1	0.71 ± 0.06	0.75 ± 0.11	0.73 ± 0.15	0.84 ± 0.25	0.82 ± 0.11	0.833	0.325	0.954
22:6 n-3	12.0 ± 4.40	13.7 ± 3.91	12.1 ± 4.34	13.4 ± 7.58	11.6 ± 3.74	0.979	0.919	0.717
∑SFA	27.9 ± 0.56	28.8 ± 0.18	28.4 ± 0.86	29.3 ± 1.80	28.8 ± 0.84	0.534	0.223	0.524
∑MUFA	28.5 ± 3.80	28.2 ± 2.72	29.6 ± 3.98	29.7 ± 5.13	30.6 ± 4.20	0.950	0.467	0.909
∑n3FA	17.4 ± 4.62	19.3 ± 4.92	17.1 ± 4.75	20.2 ± 8.92	16.5 ± 4.18	0.914	0.932	0.648
∑n6FA	25.6 ± 0.91	23.1 ± 2.34	24.1 ± 1.97	21.1 ± 6.21	23.4 ± 1.11	0.559	0.299	0.436
∑HUFA	15.7 ± 5.14	17.9 ± 5.11	15.7 ± 4.90	19.1 ± 9.39	15.3 ± 4.40	0.921	0.972	0.647
∑n3/∑n6	0.68 ± 0.20	0.85 ± 0.30	0.71 ± 0.25	1.12 ± 0.87	0.70 ± 0.18	0.921	0.972	0.647

Values are the mean of triplicate groups and are presented as a mean ± SD. Values with different superscript letters in the same row are significantly different (*p* < 0.05). The lack of superscript letters indicates no significant differences among treatments. ND, not detected. * Significance probability associated with the F-statistic.

**Table 9:** Muscle amino acid composition (%, amino acid) of red seabream (*Pagrus major*) fed the five experimental diets for 8 weeks.

						Pr > F *		
	CON	MC14	MC28	MC42	MC56	ANOVA	Linear	Quadratic
Essential amino acids								
Arg	6.42 ± 0.03	6.46 ± 0.04	6.43 ± 0.05	6.48 ± 0.07	6.43 ± 0.13	0.821	0.696	0.536
His	2.56 ± 0.05	2.51 ± 0.04	2.52 ± 0.05	2.51 ± 0.10	2.50 ± 0.05	0.712	0.238	0.614
Ile	4.60 ± 0.00	4.57 ± 0.06	4.59 ± 0.04	4.50 ± 0.08	4.52 ± 0.03	0.158	0.041	0.943
met	3.27 ± 0.07	3.31 ± 0.05	3.32 ± 0.06	3.36 ± 0.10	3.36 ± 0.03	0.441	0.083	0.635
Leu	9.29 ± 0.26	9.41 ± 0.18	9.54 ± 0.16	9.51 ± 0.32	9.50 ± 0.36	0.790	0.310	0.504
Lys	9.66 ± 0.11	9.63 ± 0.02	9.70 ± 0.13	9.64 ± 0.08	9.71 ± 0.23	0.944	0.722	0.806
Phe	4.28 ± 0.09	4.23 ± 0.11	4.28 ± 0.06	4.30 ± 0.10	4.28 ± 0.03	0.819	0.616	0.924
Thr	5.07 ± 0.04	5.03 ± 0.02	5.03 ± 0.03	5.02 ± 0.02	5.06 ± 0.05	0.464	0.877	0.088
Val	4.54 ± 0.03	4.51 ± 0.04	4.52 ± 0.02	4.50 ± 0.07	4.51 ± 0.05	0.894	0.496	0.633
Non-essential amino acids								
Ala	6.22 ± 0.11	6.25 ± 0.06	6.26 ± 0.13	6.24 ± 0.09	6.23 ± 0.09	0.987	0.938	0.597
Asp	10.2 ± 0.08	10.1 ± 0.04	10.1 ± 0.15	10.1 ± 0.27	10.1 ± 0.33	0.925	0.560	0.683
Cys	1.01 ± 0.04	1.11 ± 0.08	1.08 ± 0.08	1.05 ± 0.07	1.06 ± 0.15	0.760	0.831	0.373
Glu	15.5 ± 0.16	15.5 ± 0.08	15.5 ± 0.08	15.6 ± 0.35	16.0 ± 0.25	0.790	0.294	0.602
Gly	4.72 ± 0.14	4.86 ± 0.04	4.81 ± 0.25	4.86 ± 0.25	4.80 ± 0.23	0.905	0.675	0.513
Pro	3.42 ± 0.46	3.20 ± 0.67	3.13 ± 0.58	3.04 ± 0.71	3.06 ± 0.29	0.917	0.406	0.710
Ser	4.17 ± 0.05	4.15 ± 0.03	4.18 ± 0.00	4.27 ± 0.11	4.25 ± 0.06	0.143	0.033	0.661
Tyr	3.65 ± 0.22	3.65 ± 0.28	3.61 ± 0.18	3.69 ± 0.15	3.53 ± 0.28	0.913	0.641	0.673

Values are the mean of triplicate groups and are presented as a mean ± SD. * Significance probability associated with the F-statistic.

**Table 10:** Biometric parameters of red seabream (*Pagrus major*) fed the five experimental diets for 8 weeks.

	CF ^1^	HSI ^2^	VSI ^3^
CON	1.75 ± 0.22	1.63 ± 0.31	7.74 ± 0.88
MC14	1.66 ± 0.10	1.71 ± 0.27	7.32 ± 0.43
MC28	1.61 ± 0.10	1.83 ± 0.39	7.11 ± 0.83
MC42	1.58 ± 0.08	1.56 ± 0.27	7.11 ± 0.85
MC56	1.57 ± 0.02	1.32 ± 0.17	6.91 ± 0.66
Pr > F *			
ANOVA	0.413	0.328	0.716
Linear	0.079	0.178	0.202
Quadratic	0.494	0.127	0.695

Values are the mean of triplicate groups and presented as a mean ± SD. ^1^ Condition factor = weight (g) × 100/length^3^ (cm). ^2^ Hepatosomatic index = (liver weight (g)/fish weight (g) × 100. ^3^ Viscerosomatic index = (Viscera weight (g)/fish weight (g) × 100. * Significance probability associated with the F-statistic.

## Data Availability

The data that support the findings of this study are available from the corresponding author upon reasonable request.
